# The Complete Chloroplast Genome Sequence of *Ampelopsis*: Gene Organization, Comparative Analysis, and Phylogenetic Relationships to Other Angiosperms

**DOI:** 10.3389/fpls.2016.00341

**Published:** 2016-03-21

**Authors:** Gurusamy Raman, SeonJoo Park

**Affiliations:** Department of Life Sciences, Yeungnam University Gyeongsan, South Korea

**Keywords:** Porcelain berry, *Ampelopsis brevipedunculata*, Vitaceae, chloroplast genome, basal lineage of rosids

## Abstract

*Ampelopsis brevipedunculata* is an economically important plant that belongs to the Vitaceae family of angiosperms. The phylogenetic placement of Vitaceae is still unresolved. Recent phylogenetic studies suggested that it should be placed in various alternative families including Caryophyllaceae, asteraceae, Saxifragaceae, Dilleniaceae, or with the rest of the rosid families. However, these analyses provided weak supportive results because they were based on only one of several genes. Accordingly, complete chloroplast genome sequences are required to resolve the phylogenetic relationships among angiosperms. Recent phylogenetic analyses based on the complete chloroplast genome sequence suggested strong support for the position of Vitaceae as the earliest diverging lineage of rosids and placed it as a sister to the remaining rosids. These studies also revealed relationships among several major lineages of angiosperms; however, they highlighted the significance of taxon sampling for obtaining accurate phylogenies. In the present study, we sequenced the complete chloroplast genome of *A. brevipedunculata* and used these data to assess the relationships among 32 angiosperms, including 18 taxa of rosids. The *Ampelopsis* chloroplast genome is 161,090 bp in length, and includes a pair of inverted repeats of 26,394 bp that are separated by small and large single copy regions of 19,036 bp and 89,266 bp, respectively. The gene content and order of *Ampelopsis* is identical to many other unrearranged angiosperm chloroplast genomes, including *Vitis* and tobacco. A phylogenetic tree constructed based on 70 protein-coding genes of 33 angiosperms showed that both Saxifragales and Vitaceae diverged from the rosid clade and formed two clades with 100% bootstrap value. The position of the Vitaceae is sister to Saxifragales, and both are the basal and earliest diverging lineages. Moreover, Saxifragales forms a sister clade to Vitaceae of rosids. Overall, the results of this study will contribute to better support of the evolution, molecular biology and genetic improvement of the plant *Ampelopsis*.

## Introduction

Flowering plants are the largest clade in the land plants, containing more than 250,000 species (Friis et al., 2006). Within the land plants, the eudicot clade comprises approximately 75% of all flowering plant species, including several major subclades; namely, rosids, asterids, Saxifragales, Santalales, and Caryophyllales (The angiosperm phylogeny group II [APG II], 2003; Judd and Olmstead, 2004; Soltis et al., 2005). Among these, rosid is the largest major clade of core eudicots, comprising 140 families and 70,000 species that include nearly one third of all angiosperms (Magallón-Puebla et al., 1999; Soltis et al., 2005; Jansen et al., 2006). Due to rapid radiation, angiosperms show extraordinary diversity in habit, morphology, anatomy, physiology, and reproductive biology (Friis et al., 2006). This distinction in flowering plants has offered key challenges to evolutionary biologists investigating the origin and evolution of their traits, and determining these issues precisely depends on having a well agreed upon and strongly supported phylogenetic framework. Over the past three decades, several morphological and molecular phylogenetic studies have been used to access the relationships among the major clades, resulting in angiosperms being classified into 59 orders and 413 families (The angiosperm phylogeny group III [APG III], 2009).

In earlier studies, molecular phylogenetic analyses of flowering plants were analyzed based on one to several genes of the chloroplast (cp), mitochondrial, and nuclear genomes, but most of this analysis was based on chloroplast marker genes (Jansen et al., 2006). The relationships among many of the major lineages of angiosperms have been resolved by these efforts; however, the relationship between angiosperms and seed plants are still unclear (Friis et al., 2006). Burleigh and Mathews (2004) reported that based on rootings of phylogenetic tree analysis of DNA sequences data suggested that angiosperms are sister group to all other seed plants, whereas, *Ginkgo* and cycads separate angiosperm groups. Though, it is not straight forward to identify the position of the root in this tree. So, this pattern of relationships is difficult to interpret in evolutionary terms as it conflicts with stratigraphic evidence (Burleigh and Mathews, 2004). The complete cp genome sequence analyses have resolved problematic deep level relationships in the angiosperms (Goremykin et al., 2003, 2004, 2005; Leebens-Mack et al., 2005; Chang et al., 2006; Jansen et al., 2006), implying that Chloranthaceae and magnoliids are sister to a clade of monocots and eudicots plus Ceratophyllaceae (Jansen et al., 2007; Moore et al., 2007). Similarly, improvements have been made in elucidating relationships within the larger monocot (Graham et al., 2006) and asterid (Bremer et al., 2002) clades. Also, cp genome sequences have been widely used for plant identification, phylogenetic studies and to increase phylogenetic resolution at low taxonomic levels (Parks et al., 2009). Hence, there is rising interest in increasing the analysis of complete chloroplast genome sequences and emerging evolutionary models for phylogenetic analysis of cp sequences to address these problems (Ané et al., 2005; Jansen et al., 2006).

Despite these achievements, the position of rosids is still the least resolved major clade in the angiosperms (Soltis and Soltis, 2004). The relationships among Vitaceae are indistinct in angiosperms, and the family does not appear to have any close relatives to other families of rosids (Soltis et al., 2000). In the Cronquist system, the Vitaceae family was placed near Rhamnaceae (Cronquist, 1988). Previous studies also reported that basal nodes with the core eudicot clade have constantly received low internal support (Judd and Olmstead, 2004; Soltis et al., 2005, 2007; Schönenberger and von Balthazar, 2006), and the phylogenetic position of Vitaceae has been controversial for many years. Earlier molecular phylogenetic analyses were conducted using one to four genes, suggesting weak support for the placement of Vitaceae as a sister to asterids (Chase et al., 1993), Caryophylales (Chase et al., 1993), Dilleniaceae (Hilu et al., 2003), rosids (Savolainen et al., 2000; Soltis et al., 2000, 2003), or Saxifragales (Savolainen et al., 2000). Moreover, the latest Angiosperm Phylogeny Group (APG) III in 2009 reported that Vitaceae has its own order, Vitales (Green and Martin, 2013). Jansen et al. (2006) reported that Vitaceae was a sister-group to all other rosids based on phylogenetic analyses. Although, analyses agree on the composition of the rosid clade, the relationships within the rosids remain unclear (Wang et al., 2009).

Here, we report the complete cp genome sequence of *Ampelopsis brevipedunculata* for the first time. In addition to describing the structure of the cp genome, we provide the comparative analyses of the cp genome sequences of its closely related species of rosids. We also present the results of phylogenetic analyses of DNA sequences for 70 protein-coding genes from *Ampelopsis* and 32 angiosperm cp genomes, including 18 in the rosids clade. The phylogenetic analyses enabled elucidation of the relationships and placement of Vitaceae to other major lineages of rosids and show the importance of taxon sampling. The complete cp genome sequence of *Ampelopsis* also provides valuable data useful to chloroplast genetic engineering of this economically important medicinal and ornamental plant.

## Materials and Methods

### DNA Extraction and Sequencing

Total genomic DNA was extracted from fresh young leaves of the *A. brevipedunculata* plant using a modified CTAB Method (Doyle and Doyle, 1990). The high quality DNA was sequenced using an Illumina NextSeq 500 (LabGenomics, South Korea). The pair-end library was constructed with an insert size of ∼101 bp. Sequence trimming, assembly and mapping were performed using Genious v7.1.9 (Biomatters, New Zealand). The chloroplast genome reads were aligned to its closest cpDNA sequence of *Vitis* (GenBank accession number: NC_007957). The consensus sequences were extracted and gaps were filled by polymerase chain reaction (PCR) amplification using specific primers based on the gap between sequences. The PCR products were purified and sequenced by conventional Sanger sequencing. The sequencing data and gene annotation were submitted to GenBank and assigned an accession number of KT831767.

### Annotation and Genome Analysis of the *Ampelopsis* Chloroplast Genome

The initial annotation of the chloroplast genome was conducted using a Dual Organeller GenoMe Annotator (DOGMA; Wyman et al., 2004). From this initial annotation, putative starts, stops, and intron positions were identified based on comparisons to homologous genes of *Vitis*, *Liquidambar*, and *Nicotiana tabacum*. Further, the identified tRNAs were confirmed with tRNAscan-SE 1.21 (Schattner et al., 2005). A circle cp genome map was drawn using the OGDRAW program (Lohse et al., 2009).

### Comparative Genome Analysis of the *Ampelopsis* cp Genome

The complete cp genome of *Ampelopsis* was compared with that of four other species, *Vitis*, *Liquidambar*, *Penthorum* and *N. tabacum*, using the mVISTA program in a Shuﬄe-LAGAN mode (Frazer et al., 2004). *Ampelopsis* was set as a reference.

### Analysis of Tandem Repeats and Single Sequence Repeats (SSRs)

The presence of tandem repeats with more than 30 bp and a minimum of 90% sequence identity was also analyzed. PHOBOS v3.3.12 was used to identify tandem repeats and single sequence repeats (SSRs). The analysis parameters of alignment scores for the match, mismatch, gap, and N positions were set as 1, -5, -5, and 0, respectively (Mayer et al., 2010).

### Synonymous (K_S_) and Non-synonymous (K_A_) Substitution Rate Analysis

The *Ampelopsis* cp genome sequence was compared with the cp genome sequences of *Vitis*, *Liquidambar*, and *Penthorum*. To analyze synonymous (K_S_) and non-synonymous (K_A_) substitution rates, the same individual functional protein-coding exons were extracted and aligned separately using Geneious v7.1.9. These aligned sequences were translated into protein sequences and analyzed. The synonymous (K_S_) and non-synonymous (K_A_) substitution rates for each protein-coding exon were estimated in DnaSP (Librado and Rozas, 2009).

### Phylogenetic Analysis

In this study, genome model have been selected based on closely related to each other families of eudicots and also from previously reported studies in the literatures. A molecular phylogenetic tree was constructed using 70 protein-coding genes of 33 angiosperms. Among these 33 taxa, *Nelumbo* was set as the outgroup. The 33 completed cp genome sequences representing the lineages of angiosperms were downloaded from the NCBI Organelle Genome Resource database (Supplementary Table S1). The 70 protein-coding gene sequences were aligned using MAFFT v7.017 (Katoh and Standley, 2013) through Geneious v7.1.9. The aligned protein-coding gene sequences were saved in PHYLIP format using Clustal X v2.1 (Larkin et al., 2007) and used to generate a phylogenetic tree. Phylogenetic analysis was conducted based on maximum likelihood (ML) analysis using the general time-reversible invariant-sites (GTRI) nucleotide substitution model with the default parameters in RAxML v. 7.2.6 (Stamatakis et al., 2008). The bootstrap probability of each branch was calculated by 1000 replications.

## Results

### *Ampelopsis* cp Genome Assembly, Organization, and Gene Content

Overall, 50,269,822 paired-end reads (101 × 2) with an insert size of ∼101 bp were constructed and 2,688,617 reads were generated using 1,646,907,308 base pairs. *De novo* assembly was performed using Geneious v7.1.9. The generated contigs were assembled using the cpDNA genome sequence of *Vitis vinifera* as a reference and gaps were filled by Sanger sequencing.

**FIGURE 1:**
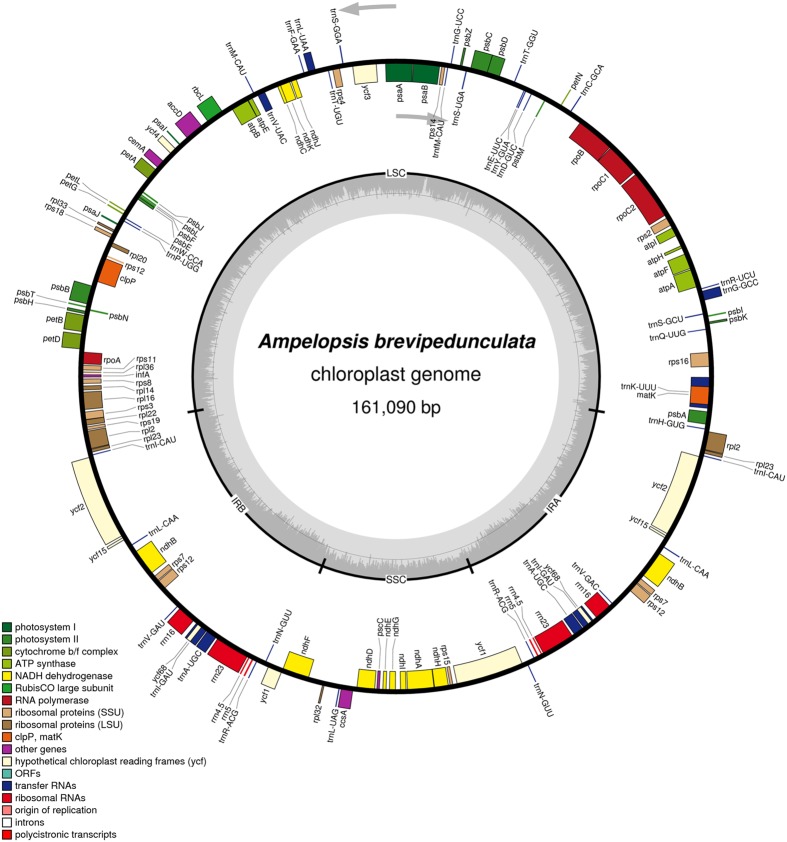
**Gene map of the *Ampelopsis brevipedunculata***. Genes lying outside of the outer layer circle are transcribed in the counterclockwise direction, whereas genes inside of this circle are transcribed in the clockwise direction. The colored bars indicate known protein-coding genes, tRNA genes and rRNA genes. The dashed darker gray area in the inner circle denotes GC content, while the lighter gray area indicates the AT content of the genome. LSC, large-single-copy; SSC, small-single-copy; IR, inverted repeat.

The complete cp genome sequence of *A. brevipedunculata* (KT831767) is 161,090 bp and shows a characteristic circular structure, including a pair of IRs (26,394 bp each) that divide the genome into two single-copy regions (LSC 89,266; SSC 19,036 bp; **Figure 1**). Coding regions (92,772 bp), comprising protein-coding genes (80,943 bp), tRNA genes (2,795 bp) and rRNA genes (9,034 bp) account for 57.59% of the genome, whereas non-coding regions (68,318 bp), including introns (16,931 bp) and intergenic spacers (51,387 bp), account for the remaining 42.41% of the genome. The overall A+T content of the whole genome is 62.6% (**Table 1**).

**Table 1:** Summary of chloroplast genome characteristics of Vitaceae.

Genome features	*Ampelopsis brevipedunculata*	*Vitis vinifera*
Size (bp)	161,090	160,928
LSC length (bp)	89,266	89,147
SSC length (bp)	19,036	19,065
IR length (bp)	26,394	26,358
Number of genes	113	113
Protein-coding genes	79+6	79+6
tRNA genes	30+7	30+7
rRNA genes	4+4	4+4
Number of genes duplicated in IR	18	18
GC content (%)	37.4	37.4

There are a total of 131 genes in the genome, including 79 protein-coding genes, 30 tRNA genes, four ribosomal RNA genes and 18 duplicated genes (**Figure 1**; **Table 2**). Of the 18 duplicated genes in the IR region, seven are protein-coding, seven are tRNA and four are rRNA genes. Eighteen genes contain introns (one class I intron, *trnL^UAA^* and 17 class II introns), and three of these genes *clpP*, *rps12*, and *ycf3*, contain two introns (**Table 3**). The 5′-end exon of the *rps12* gene is located in the LSC region, and the intron 3′-end exon of the gene is situated in the IR region. Overall, the gene order in the *Ampelopsis* chloroplast genome is identical to that of *Vitis* and tobacco.

**Table 2:** List of genes present in *Ampelopsis* chloroplast genome.

Category	Gene group	Gene name				
Self-replication	Ribosomal RNA genes	*rrn4.5*^a^	*rrn5*^a^	*rrn16*^a^	*rrn23*^a^	
	Transfer RNA genes	*trnA*-UGC^a,b^	*trnC*-GCA	*trnD*-GUC	*trnE*-UUC	*trnF*-GAA
		*trnfM*-CAU	*trnG*-GCC	*trnG*-UCC^b^	*trnH*-GUG	*trnI*-CAU^a^
		*trnI*-GAU^a,b^	*trnK*-UUU^b^	*trnL*-CAA^a^	*trnL*-UAA^b^	*trnL*-UAG
		*trnM*-CAU	*trnN*-GUU^a^	*trnP*-UGG	*trnQ*-UUG	*trnR*-ACG^a^
		*trnR*-UCU	*trnS*-GCU	*trnS*-GGA	*trnS*-UGA	*trnT*-GGU
		*trnT*-UGU	*trnV*-GAC^a^	*trnV*-GAU	*trnV*-UAC^b^	*trnW*-CCA
		*trnY*-GUA				
	Small subunit of ribosome	*rps2*	*rps3*	*rps4*	*rps7*^a^	*rps8*
		*rps11*	*rps12*^a,c,d^	*rps14*	*rps15*	*rps16*^b^
		*rps18*	*rps19*			
	Large subunit of ribosome	*rpl2*^a^	*rpl14*	*rpl16*^b^	*rpl20*	*rpl22*
		*rpl23*	*rpl32*	*rpl33*	*rpl36*	
	DNA-dependent RNA polymerase	*rpoA*	*rpoB*	*rpoC1*^b^	*rpoC2*	
	Translational initiation factor	*infA*				
Genes for photosynthesis	Subunits of photosystem I	*psaA*	*psaB*	*psaC*	*psaI*	*psaJ*
		*ycf3*^c^	*ycf4*			
	Subunits of photosystem II	*psbA*	*psbB*	*psbC*	*psbD*	*psbE*
		*psbF*	*psbH*	*psbI*	*psbJ*	*psbK*
		*psbL*	*psbM*	*psbN*	*psbT*	*psbZ*
	Subunits of cytochrome	*petA*	*petB*^b^	*petD*^b^	*petG*	*petL*
		*petN*				
	Subunits of ATP synthase	*atpA*	*atpB*	*atpE*	*atpF*^b^	*atpH*
		*atpI*				
	Large subunit of Rubisco	*rbcL*				
	Subunits of NADH dehydrogenase	*ndhA*^b^	*ndhB*^a,b^	*ndhC*	*ndhD*	*ndhE*
		*ndhF*	*ndhG*	*ndhH*	*ndhI*	*ndhJ*
		*ndhK*				
Other genes	Maturase	*matK*				
	Envelope membrane protein	*cemA*				
	Subunit of acetyl-CoA	*accD*				
	C-type cytochrome synthesis gene	*ccsA*				
	Protease	*clpP*^c^				
	Component of TIC complex	*ycf1*^a^				

*^a^Two gene copies in IRs; ^b^gene containing a single intron; ^c^gene containing two introns; ^d^gene divided into two independent transcription units*.

**Table 3:** Location and length of intron-containing genes in the *Ampelopsis* chloroplast genome.

Gene^∗^	Location	Exon I	Intron I	Exon II	Intron II	Exon III

		**Nucleotides in base pairs**				
*atpF*	LSC	144	747	414		
*clpP*	LSC	71	817	292	634	228
*ndhA*	SSC	552	1132	540		
*ndhB*	IR	777	679	756		
*petB*	LSC	6	695	642		
*petD*	LSC	8	731	475		
*rps12^#^*	LSC	114	–	232	536	26
*rpl2*	IR	390	674	435		
*rpl16*	LSC	9	1068	399		
*rpoC1*	LSC	432	763	1617		
*rps16*	LSC	40	909	236		
*trnG*-GCC	LSC	23	707	37		
*trnA*-UGC	IR	38	803	35		
*trnI*-GAU	IR	42	950	35		
*trnK*-UUU	LSC	37	2512	29		
*trnL*-UAA	LSC	37	516	50		
*trnV*-UAC	LSC	39	574	37		
*ycf3*	LSC	126	739	228	745	153

*^∗^Identical duplicate gene containing introns in the IR region are not included. ^#^The *rps12* is a *trans*-spliced gene with the 5′ end located in the LSC region and duplicated in the 3′ end in the IR regions*.

### Comparative Analysis of the *Ampelopsis* Chloroplast Genome

**FIGURE 2:**
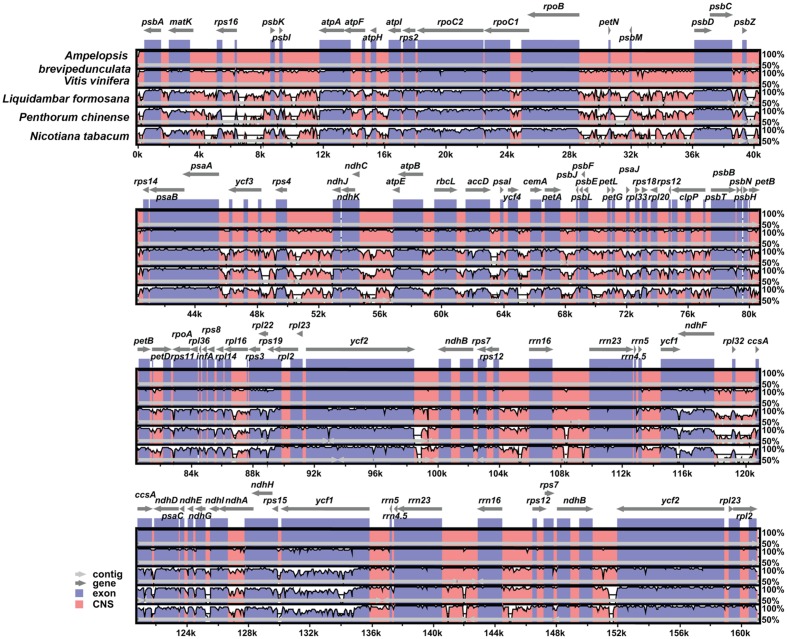
**Comparison of the cp genome sequence of *Ampelopsis brevipedunculata*, *Vitis vinfera*, *Liquidambar formosana*, *Penthorum chinense*, and *Nicotiana tabacum* generated with mVista**. Gray arrows indicate the position and direction of each gene. Red and blue areas indicate intergenic and genic regions, respectively. Black lines define regions of sequence identity with *A. brevipedunculata*, using a 50% identity cutoff. Dashed rectangles denote highly divergent regions of *A. brevipedunculata* compared to *V*. *vinfera*, *L*. *formosana*, *P*. *chinense*, and *N*. *tabacum*.

mVISTA was used to study the cp genome sequence variations in the orders of Vitales and Saxifragales, as well as in *Nicotiana*. The coding region was found to be more highly conserved than the non-coding regions (**Figure 2**), and the most dissimilar coding regions of the five chloroplast genomes were *rpl22*, *rps19*, and *ycf1*.

**FIGURE 3:**
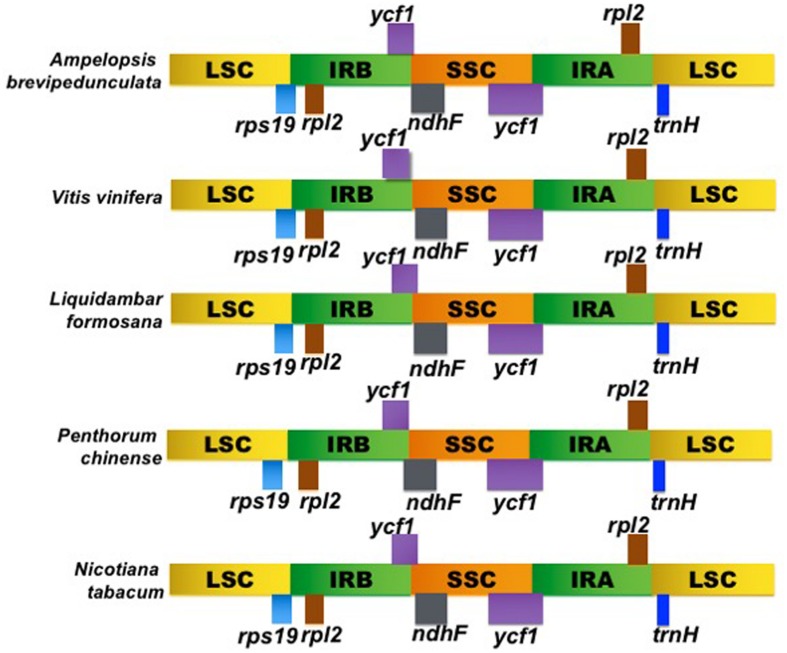
**Comparison of the borders of the LSC, SSC, and IR regions of *Ampelopsis brevipedunculata*, *Vitis vinfera*, *Liquidambar formosana*, *Penthorum chinense*, and *Nicotiana tabacum* cp genomes**.

The LSC/IRB/SSC/IRA boundary regions of the *Ampelopsis* cp genome were compared to the corresponding regions of its four closely related cp genomes, *Vitis*, *Liquidambar*, *Penthorum*, and *Nicotiana* (**Figure 3**). The *rps19* gene of *Ampelopsis*, *Vitis* and *Liquidambar* was extended from the IRB to the LSC region with 7–37 bp variability. However, the *rps19* gene of *Penthorum* and *Nicotiana* was shifted to an LSC region with a 2–15 bp gap. At the IRB/SSC boundary, the *ycf1* and *ndhF* genes were overlapped in *Ampelopsis*, *Vitis*, *Liquidambar*, *Penthorum*, and *Nicotiana*. Expansion, contraction, and shifting of the *ycf1* gene was observed in the boundary regions of the SSC/IRA. The size variation of *ycf1* from 5172 to 5682 bp was identified in all cp genomes. The *trnH* gene was located in the LSC region of all genomes, but varied from 0 to 21 bp apart from the IRA/LSC junctions. When compared with other closely related cp genomes of *Vitis*, the *Ampelopsis* was found to have very little size differences in the LSC, IR, and SSC regions.

### Repeat Sequence Analysis

**FIGURE 4:**
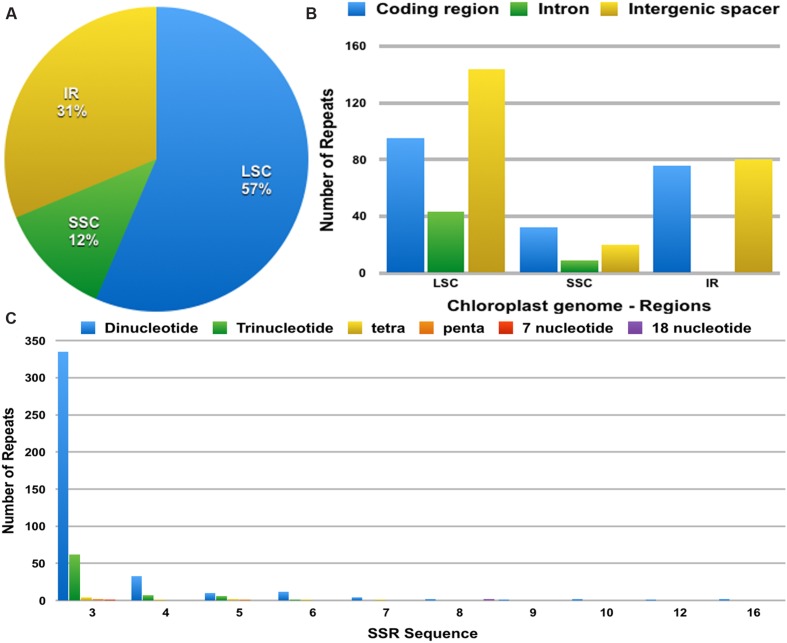
**The distribution, type and presence of simple sequence repeats (SSRs) in the cp genome of *Ampelopsis brevipedunculata*. (A)** Presence of SSRs in the LSC, SSC, and IR regions. **(B)** Presence of SSRs in the protein-coding regions, intergenic spacers and introns of LSC, SSC, and IR regions. **(C)** Presence of polymers in the cp genome of *A. brevipedunculata*.

The distribution, type and presence of simple sequence repeats (SSR) or microsatellites was studied in the cp genome of *Ampelopsis*. A total of 493 SSRs were identified (**Figure 4**). Of these, 282 were found in the LSC regions, whereas 156 and 61 were in the IR and SSC regions, respectively (**Figure 4A**). Moreover, 203 SSRs were found in the protein-coding regions, 235 were in intergenic spacers and 61 in the introns of the *Ampelopsis* cp genome (**Figure 4B**). Among these SSRs, dipolymers were most common, accounting for 81.5%, while tripolymers accounted for 15.42%, and tri-, tetra-, penta-, 7-nucleotide, and 18-nucleotide polymers occurred with less frequency (**Figure 4C**). Moreover, three penta-, one 7-nuclelotide and two 18-nucleotide polymers were detected in the cp genome. The size and location of tetra-, penta-, 7-nucleotide and 18-nucleotide polymers are shown in **Table 4**. A total of 17 polymers was identified in the genome, whereas 11 were localized in intergenic spacers, six in coding regions and none in introns.

**Table 4:** Distribution of tetra, penta, and hexapolymer single sequence repeats (SSRs) in *Ampelopsis* chloroplast genome.

SSR type	SSR sequence	SSR size (bp)	Start	End	Location
Tetra	(AAAT)3	12	52,799	52,810	*trnF*-GAA/*ndhJ* (IGS)
Tetra	(AAAT)3	12	126,739	126,750	*ndhA* (CDS)
Tetra	(AATC)3	12	127,487	127,498	*ndhA* (CDS)
Tetra	(AGAT)3	13	31,776	31,788	*petN*/*psbM* (IGS)
Tetra	(AAAG)3	13	127,046	127,058	*ndhA* (CDS)
Tetra	(AATC)3	14	68,471	68,484	*petA*/*psbJ* (IGS)
Tetra	(AGAT)4	17	1627	1643	*psbA*/*matK* (IGS)
Tetra	(AAAT)5	22	104,318	104,339	*rps12*/*trnV*-GAU (IGS)
Tetra	(AATT)5	23	54,955	54,977	*ndhC*/*trnV*-UAC (IGS)
Tetra	(AAAT)6	26	146,014	146,039	*trnV*-GAC/*rps12* (IGS)
Tetra	(AAAG)7	29	47,086	47,114	*ycf3* (CDS)
Penta	(AATAT)3	15	55,700	55,714	*ndhC*/*trnV*-UAC (IGS)
Penta	(AAAAT)3	15	70,508	70,522	*psbE*/*petL* (IGS)
Penta	(AATAT)5	27	31,699	31,725	*petN*/*psbM* (IGS)
7-nucleotide	(AAAAAAT)3	21	14,750	14,770	*atpF*/*atpH* (IGS)
18-nucleotide	(AATATCGTCACTAGCATC)	78	96,562	96,639	*ycf2* (CDS)
18-nucleotide	(AATATCGTCACTAGCATC)	78	153,718	153,795	*ycf2* (CDS)

A total of 11 tandem repeats were identified in the cp genome of *Ampelopsis* (**Table 5**). Of these, six were present in the intergenic spacers of *atpH*/*atpI* (1), *rpoB*/*trnC*-GCA (1), *psbM*/*trnD*-GUA (1), *trnE*-UUC/*trnT*-GGU (1), *psaA*/*ycf3* (1) and *ndhF*-*rpl32* (1), three were located in the protein-coding regions of *accD* (1) and *ycf2* (2) and two were present in the intron and exon of *ycf3*.

**Table 5:** Distribution of tandem repeats in *Ampelopsis* chloroplast genome.

S. no.	Repeat length (bp)	Consensus size × copy number	Start	End	Location
1	30	15 × 2	15,791	15,832	*atpH*/*atpI* (IGS)
2	40	20 × 2	28,961	29,000	*rpoB*/*trnC*-GCA (IGS)
3	45	21 × 2	32,738	32,782	*psbM*/*trnD*-GUA (IGS)
4	40	16 × 2	34,723	34,762	*trnE*-UUC/*trnT*-GGU (IGS)
5	30	15 × 2	45,933	45,962	*psaA*/*ycf3* (IGS)
6	35	16 × 2	47,890	47,924	*ycf3* (exon and intron)
7	35	17 × 2	61,967	62,014	*accD* (CDS)
8	48	24 × 2	46,377	46,406	*ycf3* (exon and intron)
9	78	18 × 4	96,562	96,639	*ycf2* (CDS)
10	37	17 × 2	119,000	119,036	*ndhF*-*rpl32* (IGS)
11	78	18 × 4	153,718	153,795	*ycf2* (CDS)

### Synonymous (K_S_) and Non-synonymous (K_A_) Substitution Rate Analysis

**FIGURE 5:**
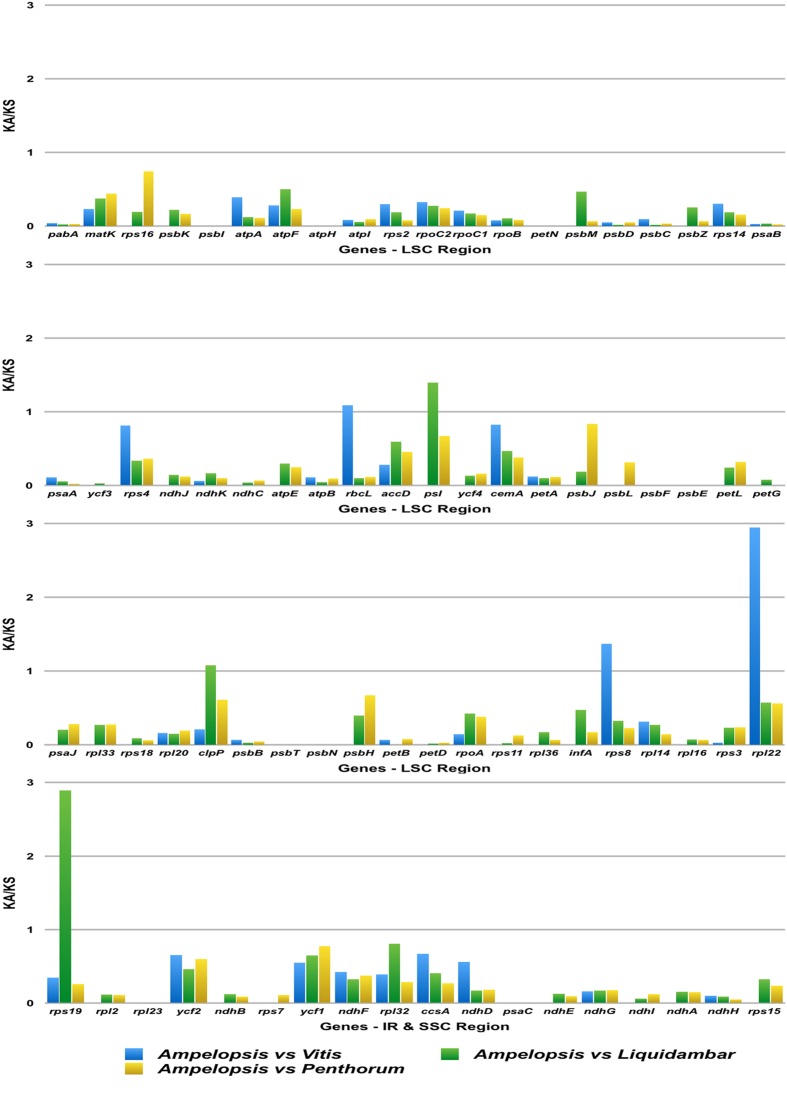
**K_A_/K_S_ values of 78 protein-coding genes of *Ampelopsis brevipedunculata*, *Vitis vinfera*, *Liquidambar formosana*, and *Penthorum chinense***. Blue color boxes indicate the K_A_/K_S_ ratio of the *A. brevipedunculata* vs. *V*. *vinfera*, Green color boxes indicate *A. brevipedunculata* vs. *L*. *formosana* and Yellow color boxes indicate *A. brevipedunculata* vs. *P*. *chinense*.

A total of 78 genes encoding 91 protein-coding exons in the cp genome of *Ampelopsis* were used to analyze synonymous and non-synonymous rates against *Vitis*, *Liquidambar*, and *Penthorum* (**Figure 5**). The K_A_/K_S_ ratio of all genes was less than 1, except for *rpl22* of *Lychnis*. The K_A_/K_S_ ratio of *rpl22* of *Ampelopsis* vs. *Vitis* was 2.95, while *rps19* of *Ampelopsis* vs. *Liquidambar* was 2.89.

### Phylogenetic Analysis

**FIGURE 6:**
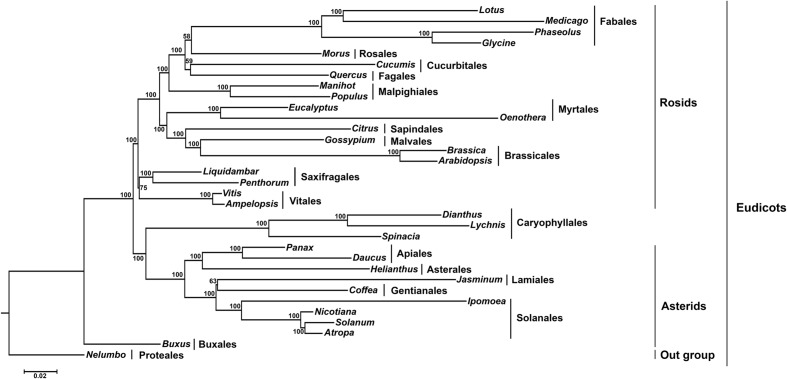
**Molecular phylogenetic tree of 33 angiosperms based on 70 protein-coding genes in the cp genome**. The tree was constructed by maximum likelihood (ML) analysis of the conserved regions using the RaxML program and the GTR+I nucleotide model. The stability of each tree node was tested by bootstrap analysis with 1000 replicates. Bootstrap values are indicated on the branches, and the branch length reflects the estimated number of substitutions per 1000 sites. *Nelumbo* was set as the outgroup.

In this study, we analyzed the relationship between Vitales and Rosids. The reconstructed phylogeny showed that it divided into two clades, rosids, and asterids (**Figure 6**). Within asterids, Caryophyllales (core eudicots) deviated from asterids and formed two sister clades with a 100% bootstrap (BS) value. In another major rosid clade, Saxifragales, and Vitaceae diverged from rosids and formed two sister clades with 100% BS value. These two clades are the earliest diverging lineages of rosids, and Saxifragales forms a sister clade to *Ampelopsis* and *Vitis* (Vitaceae) with 75% BS value.

## Discussion

Most angiosperms commonly encode 74 protein-coding genes, while an additional five are present in only some species (Millen et al., 2001). However, the *Ampelopsis* cp genome has 79 protein-coding genes, 30 tRNA genes and four ribosomal RNA genes, which is similar to *Vitis* and Saxifragales. This might have been because the genome shares its gene contents with the Vitaceae family and its closest relative, the Saxifragales family. Moreover, the total numbers of introns in the plastid are the same in this family and Saxifragales. Several lineages of angiosperms have lost introns from the *rpl2* gene independently (Downie et al., 1991), which could also be considered a characteristic feature of the core members of Caryophyllales (Logacheva et al., 2008). However, the Vitaceae family has not lost any introns in the genes.

The cp genome size of *Ampelopsis* was compared with that of *V. vinifera*. Both genomes showed a similar genome size. The detected variation in sequence length between these two genomes is only 162 bp, which might be due to the insertion of sequences in the non-coding region of *Ampelopsis*. Among plant lineages, the genome size varies due to the expansion and contraction of the border regions between the IR regions and the single-copy regions (Wang and Messing, 2011). Hence, in this study, the exact IR border positions and their adjacent genes of one Vitaceae, two Saxifrageles and one *Nicotiana* cp genomes were compared. The organization of the *Ampelopsis* genome with a pair of IR regions separated by the SSC and LSC regions is identical to most sequenced angiosperm chloroplast genomes. The photosynthetic dicot cp genome size of angiosperms varies from 150,519 bp (Lotus; Kato et al., 2000) to 162,686 bp (Amborella; Goremykin et al., 2003), whereas the size of the *Ampelopsis* cp genome (161,090 bp) is also within this size range. The size of the IR region (26,394 bp) of the *Ampelopsis* is also well within the size range of other sequenced dicot genomes that range from 23,302 (Calycanthus; Goremykin et al., 2003) to 27,807 bp (Oenothera; Hupfer et al., 2000). However, the gene content and order of the *Ampelopsis* cp genome is exactly the same as that of *Vitis*, tobacco and many other unreorganized angiosperm cp genomes. Previous studies also reported that several rosid cp genomes have lost the *rpl22* gene, including legumes (Spielmann et al., 1988; Milligan et al., 1989; Gantt et al., 1991; Doyle et al., 1995; Saski et al., 2005). At least two independent losses of *rpl22* have occurred in the rosids (Jansen et al., 2006). However, multiple independent genes *infA* (Millen et al., 2001), *rps16* (Doyle et al., 1995), and *accD* (Downie and Palmer, 1992; Cosner et al., 1997) have also been lost from the angiosperms. Taken together, these results indicate that gene losses are not always dependable markers of phylogenetic relationships.

Remarkably, the ACG start codon was found in *ndhD* and *psbL* of the *Ampelopsis* cp genome. Earlier studies also showed that, due to RNA editing during the translation process, the ACG start codon of *rps19* has been converted into an initiation codon, AUG, in *Nicotiana* and *Dianthus* (Neckermann et al., 1994; Raman and Park, 2015). The same process might also have occurred in these two genes of the *Ampelopsis* cp genome. This evidence indicates that the evolutionary rates of cp genomes in Vitaceae are comparatively mild based on the relatively minor variations in the IR regions. High sequence polymorphisms are frequently observed in closely related species of land plants and considered as it is highly conserved regions in the chloroplast genome (Wicke et al., 2011). The occurrence of several SSR sites in the *Ampelopsis* cp genome showed that these sites can be used to estimate the intraspecific level of polymorphism leading to very sensitive phylogeographic and population structure studies of this species.

The synonymous and non-synonymous nucleotide substitution patterns are very important markers in gene evolution studies (Kimura, 1983). Non-synonymous nucleotide substitutions have occurred less frequently than synonymous substitutions, and the ratio of K_A_/K_S_ was less than one in most protein-coding regions (Makalowski and Boguski, 1998). In this study, the ratio of K_A_/K_S_ was significantly less than one in all protein-coding regions of *Ampelopsis*, except for two genes. Nevertheless, the K_A_/K_S_ ratio of *rpl22 Ampelopsis* vs. *Vitis* and *rps19* of *Ampelopsis* vs. *Liquidambar* was 2.95 and 2.89, respectively. When compared with gene *rpl22 Ampelopsis* vs. *Vitis*, 10 amino acids were changed, as were nine amino acids in *rps19* of *Ampelopsis* vs. *Liquidambar*. Most of the changes occurred in the second and third position of the codon rather than the first position. This fluctuation might have been due to non-synonymous substitution in the *rpl22* and *rps19* genes and is the result of silent mutation.

The eudicots are considered the largest clade of angiosperms, containing over 75% of the extant species (Soltis et al., 2005). Within eudicots, Nelumbo diverges first and forms a clade with Buxus. Buxus is a sister to a strongly supported eudicot clade that includes two discreetly to well-supported groups encompassing the rosids and asterids. Previous studies have clearly indicated that Ranunculus, proteales and Buxales are early diverging lineages of eudicots (Friis et al., 2011). While, recent molecular phylogenetic analysis revealed that Caryophyllales belongs to sister relationship with asterids (Jansen et al., 2006; Raman and Park, 2015).

In core eudicots, the rosid clade is well-supported, but the least resolved major clade, comprising more than 25% of all angiosperm species (Schönenberger and von Balthazar, 2006). Soltis et al. (2003) reported that Saxifragales are generally associated with rosids, though support is not high and the order has been linked with caryophyllids. Saxifragales are clearly the earliest diverging lineage of core eudicots (Soltis et al., 2005; Magallon, 2007). Recently, based on 24 plastid inverted repeats, 10 plastid, and 2 nuclear genes analysis clarified the internal relationships of rosids. This study suggested that rosids formed two major clades, Fabidae and Malvidae, and the addition of Saxifragales as a basal order and Vitaceae as sister to all other rosid clade (Wang et al., 2009). Based on these considerations, phylogenetic analysis was conducted based on 70 protein-coding genes of 33 angiosperms to understand the position of *Ampelopsis* in the eudicots. Molecular phylogenetic analysis showed that both Saxifragales and Vitaceae diverged from rosids and formed two separate clades within rosids with 100% BS value. Vitals (*Ampelopsis* and *Vitis*) are a sister clade to Saxifragales with a 75% moderate bootstrap value. However, according to The angiosperm phylogeny group III [APG III] (2009), the placement of Vitaceae is in its own order, while Vitales is in the eudicots (Green and Martin, 2013). Although phylogenetic studies support that Vitaceae is an early diverging member and forms a sister-group to all other rosids (Jansen et al., 2006), the analysis conducted in this study shows that Saxifragales and Vitaceae are the ancient early diverging members of the rosid clade and Saxifragales formed a sister relationship with Viataceae. The phylogenetic analysis based on these results strongly supports that Vitaceae is a sister to Saxifragales and that both are early diverging clades within the rosids. However, the relationship of Vitaceae with Saxifragales is equivocal.

## Conclusion

In summary, the chloroplast genome of *Ampelopsis* was sequenced and characterized for the first time. The *Ampelopsis* genome shares the same overall organization and gene contents of most of the unreorganized angiosperm chloroplast genomes, including its closest species, *Vitis*. The LSC/IRB/SSC/IRA boundary regions of the *Ampelopsis* cp genome were compared to its closely related genomes and no intense variations were identified in Vitaceae. A phylogenetic tree constructed with 70 protein-coding genes of 33 angiosperms revealed strong support for the position of Saxifragales and Vitaceae as the basal and earliest diverging lineages. Moreover, the analysis indicated that Saxifragales forms a sister to Vitaceae of the rosids. Overall, the results of this study provide better support of the evolution and molecular biology of the plant, *Ampelopsis*, and will enable its genetic improvement.

## Author Contributions

Conceived and designed the experiments: SP. Performed the experiments, analyzed the data, and wrote the paper: GR.

## Conflict of Interest Statement

The authors declare that the research was conducted in the absence of any commercial or financial relationships that could be construed as a potential conflict of interest.
