# Corrigendum: The Complete Chloroplast Genome Sequence of *Ampelopsis*: Gene Organization, Comparative Analysis, and Phylogenetic Relationships to Other Angiosperms

**DOI:** 10.3389/fpls.2018.01821

**Published:** 2018-12-13

**Authors:** Gurusamy Raman, SeonJoo Park

**Affiliations:** Department of Life Sciences, Yeungnam University, Gyeongsan, South Korea

**Keywords:** Porcelain berry, *Ampelopsis brevipedunculata*, Vitaceae, chloroplast genome, basal lineage of rosids

There is an error in the Funding. The correct name for the funder is “The National Institute of Biological Resources of Korea”. Website: https://www.nibr.go.kr. In addition, the correct number for The National Institute of Biological Resources of Korea is “NIBR201631201”.

The authors apologize for this error and state that this does not change the scientific conclusions of the article in any way. The original article has been updated.

